# Early identification of glufosinate-resistant soybeans using plant image analysis

**DOI:** 10.3389/fpls.2026.1886688

**Published:** 2026-08-04

**Authors:** Min-Jung Yook, Bo-Kyung Chang, Tae-Kyeong Noh, Do-Soon Kim

**Affiliations:** Department of Agriculture, Forestry and Bioresources, Research Institute of Agriculture and Life Sciences, College of Agriculture and Life Sciences, Seoul National University, Seoul, Republic of Korea

**Keywords:** chlorophyll fluorescence, herbicide resistance, high-throughput screening, machine learning, non-destructive phenotyping, spectral imaging

## Abstract

The use of glufosinate-resistant GM soybean has expanded, raising concerns about resistant weed development and unintended transgene flow. To support monitoring for timely management, we propose an early, non-destructive identification method using spectral images acquired from whole soybean plants after glufosinate treatment. We evaluated the potential of spectral imaging, using RGB, infrared (IR) thermal, and chlorophyll fluorescence (CF) sensors, for early detection of glufosinate resistance in soybean. In the dose-response test, the key spectral indices including NDI, temperature difference, F_v_/F_m_, and NPQ distinguished between resistant and susceptible soybeans within 4 to 24 hours after treatment (HAT). IR thermal and CF imaging showed higher sensitivity in identifying resistance than RGB imaging by detecting spectral responses associated with physiological changes before visual symptoms appeared. Validation test with a single dose treatment of glufosinate reconfirmed that image analysis by both the naked eye and machine learning (ML) can discriminate between resistant and susceptible soybeans in a single day after glufosinate treatment. ML-based classification using IR thermal index achieved 100% accuracy as early as 6 HAT and the classification by the naked eye using IR thermal images showed 96.6% accuracy at 24 HAT. These results suggest that plant imaging enables early and non-destructive identification of herbicide-resistant individuals by detecting early spectral changes to herbicide treatment. These findings support its use as a potential alternative to conventional diagnostic methods for detecting individuals containing transgenes in herbicide-resistant GM soybean cultivation for future applications in herbicide-resistant weed monitoring.

## 1 Introduction

The use of glufosinate in herbicide-resistant (tolerant) GM crop cultivation has expanded to manage glyphosate-resistant weeds, either as an alternative to glyphosate or in combination with it through stacked-trait cultivars (Beckie, 2011; Heap and Duke, 2018). The adoption of glufosinate-resistant soybean cultivars, which carry either the *pat* or *bar* gene encoding phosphinothricin acetyltransferase (PAT), has facilitated the use of glufosinate in soybean production (Herouet et al., 2005). Although the number is lower than that of glyphosate resistance, eleven cases of glufosinate-resistant weeds have been reported in different cropping systems so far (Heap, 2026). This suggests that the development of glufosinate-resistant weeds may become an increasing concern if the cultivation of glufosinate-resistant GM crops expands and results in greater use of glufosinate. In addition, the cultivation of glufosinate-resistant soybean also raises concerns about the unintentional contamination of off-target soybean plants through transgene flow (Price and Cotter, 2014; Yook et al., 2021). Considering these two major concerns on glufosinate-resistant weeds and transgene flow, early and rapid monitoring of glufosinate resistance is essential for proactive management.

For monitoring herbicide resistance in weeds and crops, the whole-plant assay is conventionally the most common diagnostic method. However, this is time-consuming, labor-intensive, and requires large space (Song et al., 2017; Lim et al., 2021). Various alternative diagnostic methods including growth pouch, trimmed stem node, and leaf disc tests have been developed for resistance diagnosis (Zhang et al., 2015; Kim et al., 2000; Koger et al., 2005). However, these methods are difficult to apply under actual field conditions because they are often destructive and restricted to specific plant parts. To overcome these limitations and provide alternatives to conventional diagnosis, spectral image-based approaches have been attempted, by using images acquired across a wide range of wavelengths, including both visible and non-visible regions. For diagnosing plant conditions, RGB, infrared (IR) thermal, and chlorophyll fluorescence (CF) images are regarded as highly effective tools (Noh and Kim, 2018). These techniques allow for the non-destructive and rapid evaluation of plant responses to stressors, making them useful for identifying herbicide induced changes, such as morphological changes, photosynthetic inhibition, and alterations in leaf temperature (Weber et al., 2017; Marques et al., 2021; Pineda et al., 2021; Noh et al., 2025).

More recently, studies on herbicide resistance have combined hyperspectral imaging (HSI) with machine learning or deep learning approaches. For example, HSI combined with machine learning has been used to distinguish glyphosate-resistant and susceptible johnsongrass and to evaluate herbicide safener effects in wheat (Huang et al., 2022; Chu et al., 2025). UAV-based multimodal imaging and deep learning have also been applied to field-scale weed resistance assessment, and HSI combined with deep learning has been used for early prediction of herbicide resistance in maize (Xia et al., 2023; Xiao et al., 2024). However, HSI-based approaches require high-dimensional spectral data processing and complex analysis workflows, which may limit their practical application (Garcia-Salgado et al., 2020). Therefore, simple image-based approaches using RGB, IR thermal, and chlorophyll fluorescence imaging remain a useful option for rapid screening and monitoring because they involve relatively simple analysis procedures and provide indices that are readily interpretable in relation to plant physiological responses.

Despite this potential, using spectral imaging to detect glufosinate resistance in soybean has rarely been explored. Jones et al. (2023) provided a proof-of-concept using a multispectral sensor combined with a leaf disc assay at a range of glufosinate concentrations. However, further validation using whole-plant and under practical conditions has not been done so far. Therefore, this study aimed to develop an early, non-destructive diagnostic method for the identification of glufosinate-resistant soybeans using spectral imaging. We evaluated the spectral responses of whole soybean plants treated with different doses of glufosinate and identified key spectral indices derived from RGB, IR thermal, and CF images for early discrimination between glufosinate-resistant and susceptible soybeans. Furthermore, we validated the classification accuracy of the selected indices in mixed plantings of resistant and susceptible soybeans, confirming their reproducibility and potential applicability. By enabling early detection of glufosinate resistance, this approach can be applied to monitor transgene flow from glufosinate-resistant GM soybean and could be expanded to monitor herbicide-resistant weeds.

## 2 Materials and methods

**Figure 1:**
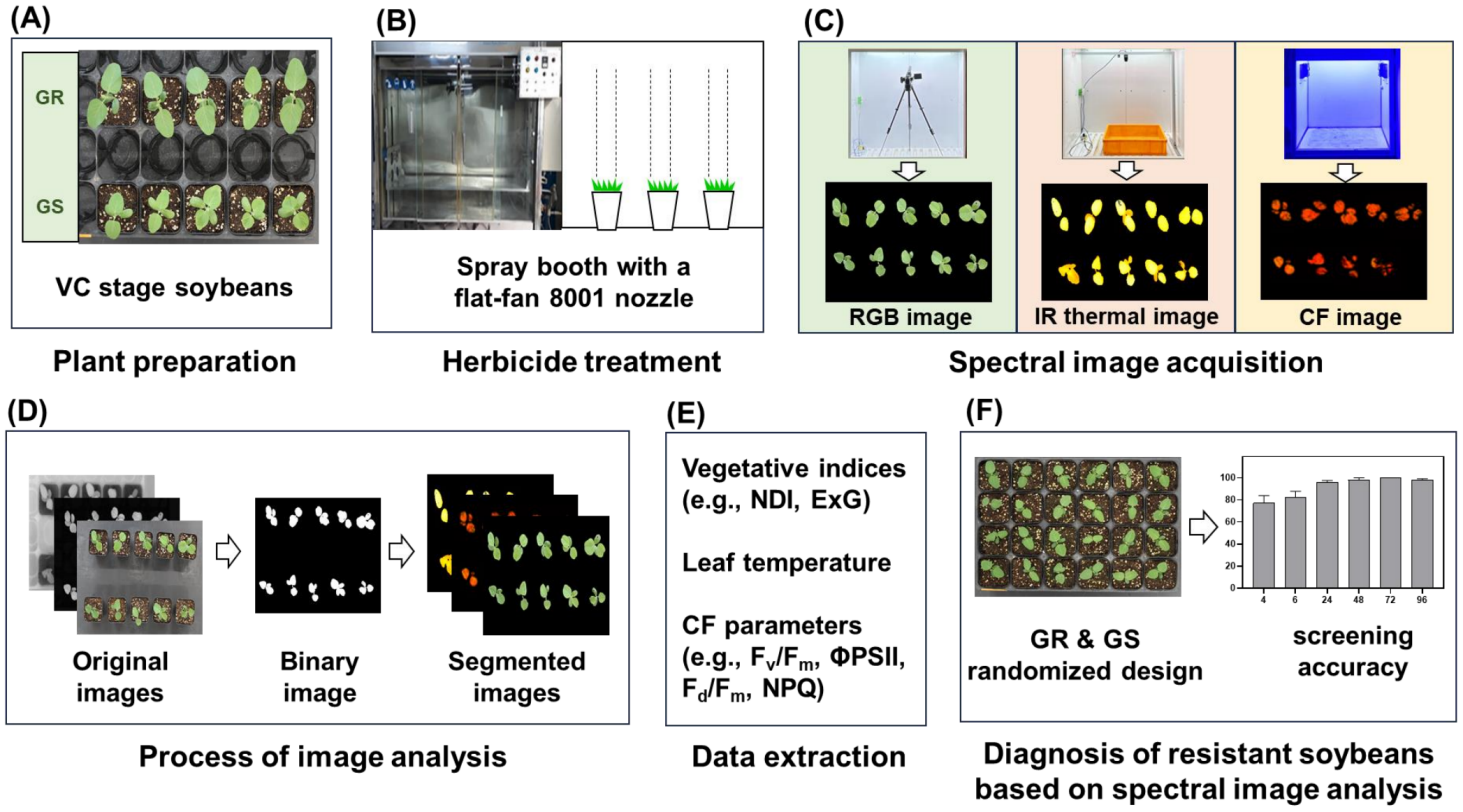
The overall workflow for identification of glufosinate-resistant soybeans using spectral image analysis: **(A)** plant preparation, **(B)** herbicide treatment, **(C)** spectral image acquisition, **(D)** image processing, **(E)** data extraction, and **(F)** diagnosis of glufosinate-resistant individuals. GR, glufosinate-resistant soybean; GS, glufosinate-susceptible soybean; VC, cotyledon stage; RGB, red-green-blue; IR, infrared; CF, chlorophyll fluorescence; NDI, normalized difference index; ExG, excess green index; F_v_/F_m_, maximum quantum yield; Φ_PSII_, PSII quantum yield; Fd/F_m_, chlorophyll fluorescence decrease ratio; NPQ, non-photochemical quenching.

This study consisted of two experiments (**Figure 1**). First, a dose-response test was carried out to evaluate spectral changes in glufosinate-resistant and susceptible soybeans after herbicide treatment. Second, a validation test was conducted to confirm the reproducibility and potential applicability of spectral indices identified in the first test, by assessing their classification accuracy between resistant and susceptible soybeans using plant image analysis.

### 2.1 Plant materials

For the dose-response test, a glufosinate-resistant GM soybean (AtSIZ #6, *Glycine max* L. cv. Kwangankong) and its non-transgenic maternal parent ‘Kwangankong’ were used as the resistant and susceptible varieties, respectively. A total of 40 plants were used, including four replicates for each combination of soybean variety and glufosinate dose. Plant images of the same plants were taken repeatedly after glufosinate treatment. For the validation test, F_2_ hybrid population containing both resistant and susceptible individuals derived from crosses between GM and wild soybean (*G. soja* Sieb. and Zucc., IT 182932) was used. A total of 96 F_2_ plants were used, with 24 individuals in each of four replicates. These plant materials were established as described by Yook et al. (2021). All soybean seedlings were grown in a growth chamber (HB-301SP-0, Hanbaek Science, Korea) maintained at 26 ± 3°C with a 12 h photoperiod.

### 2.2 Herbicide treatment

For the dose-response test, glufosinate-ammonium (Basta^®^, Bayer Crop Science Ltd., Korea; hereinafter glufosinate) was applied to the foliage of soybean plants at the VC stage. Glufosinate doses tested were 90, 180, 360, and 720 g a.i. ha^-1^, which correspond to 1/16× to 1/2× of the recommended dose (1440 g a.i. ha^-1^) and an untreated control was included. For the validation test, a single dose of 180 g a.i. ha^-1^ was applied, as it was the minimum dose that differentiated resistant and susceptible soybeans across all spectral indices in the dose-response test. Dose selection was based on the overall discrimination patterns observed across image types and assessment time points rather than on the results from a single assessment time point. Glufosinate was applied using a compressor-pressurized belt-driven sprayer (R&D Sprayers, USA) equipped with an 8001 flat-fan nozzle (Spraying Systems Co., USA), delivering a spray volume of 500 L ha^-1^. After herbicide treatment, the soybean plants were immediately transferred to the growth chamber and arranged in a randomized block design with four replicates.

### 2.3 Spectral image acquisition and analysis

For the dose-response test, spectral images were taken at 4, 6, 24, 48, 72, and 96 hours after glufosinate treatment (HAT), with the 6 HAT time point acquired during the night period. In addition, aboveground biomass (fresh weight) was measured 10 days after treatment under each dose condition to support the spectral analysis. Biomass was measured by destructively harvesting the same four plants repeatedly imaged in each treatment. For the validation test, images were taken at 4, 6, 24, and 48 HAT during the light period. The acquisition and processing methods for each type of spectral image are described below.

#### 2.3.1 RGB images

RGB images of soybean plants were acquired using a CMOS camera (EOS-600D, Canon, Japan) mounted vertically at a fixed height of 80 cm above the plant canopy. Images were captured under controlled light conditions to minimize variations caused by ambient light. The camera settings were fixed throughout the experiment (ISO 1600, shutter speed 1/60 s, aperture f/4.5, focal length 33 mm, and image resolution 5184 × 3456 pixels). At this imaging distance, the ground sampling distance (GSD) corresponded to approximately 0.13 mm pixel^-1^. The images were processed with MATLAB 2021b (The MathWorks Inc., USA). To segment the plant area, the RGB images were converted into Lab color space, and background removal was performed using thresholding. The greenness of soybean leaves was assessed using the normalized difference index (NDI) and the excess green index (ExG) as follows (Equation 1–3):

(1) $$\text{r}=\;\frac{R}{R+G+B},\text{ g}=\;\frac{G}{R+G+B},\text{ b}=\frac{B}{R+G+B}$$

(2) $$\text{NDI}=\;\frac{g-r}{g+r}$$

(3) ExG=2g−r−b

where R, G, and B represent the original red, green, and blue pixel values, respectively, and r, g, and b represent the corresponding normalized values.

#### 2.3.2 IR thermal images

IR thermal images of soybean plants were captured using a radiometrically calibrated IR thermal camera (A65sc, FLIR, Sweden) mounted inside a growth chamber maintained at 33°C. The IR thermal camera has a resolution of 640 × 512 pixels and was positioned at a fixed distance of 60 cm from the plant canopy. The images were processed and analyzed with MATLAB 2021b. To isolate plant-specific data, the IR thermal images were registered with the corresponding RGB images, images acquired from the same viewing angle. Image registration was performed using manually selected corresponding control points between RGB and IR thermal images, followed by geometric transformation and alignment in MATLAB. After registration, plant regions segmented from the RGB images were used as masks to remove background pixels from the IR thermal images, enabling extraction of leaf temperature values only from soybean tissues. The temperature difference between glufosinate-treated and untreated plants was calculated using the following formula (Equation 4),

(4) $$\text{Temperature difference}=\;T_{h}-T_{0}$$

where *T_h_* and *T_0_* indicate leaf temperatures (°C) of glufosinate-treated and untreated soybean plants, respectively.

#### 2.3.3 CF images

CF images of soybean plants were acquired using the SNU-KIST 2 imaging system (Seoul National University/KIST, Korea), with the camera positioned at a fixed distance of 60 cm above the plant canopy. The plants were dark-adapted for 30 minutes prior to image acquisition. For each CF image, the pixel intensity of soybean plants was extracted and averaged to derive the CF value, with the background removed using Otsu thresholding. Four CF indices were used in this study: maximum quantum yield (F_v_/F_m_), PSII quantum yield (Φ_PSII_), chlorophyll fluorescence decrease ratio (F_d_/F_m_), and non-photochemical quenching (NPQ). These indices were calculated using the following formulae (Equations 5–8),

(5) $${\text{F}}_{\text{v}}/{\text{F}}_{\text{m}}=\frac{F_{m}-F_{0}}{F_{m}}$$

(6) $${\text{Φ}}_{\text{PSII}}=\frac{{F^{\prime }}_{m}-F_{s}}{{F^{\prime }}_{m}}$$

(7) $${\text{F}}_{\text{d}}/{\text{F}}_{\text{m}}=\frac{F_{m}-{F^{\prime }}_{m}}{F_{m}}$$

(8) $$\text{NPQ}=\frac{F_{m}-{F^{\prime }}_{m}}{{F^{\prime }}_{m}}$$

where *F_0_* is the ground CF at dark-adapted state, *F_m_* is the CF value at 1 second when a plant emits maximum fluorescence, and *F′_m_* is the CF value at maximum fluorescence intensity measured at the light-adapted state.

### 2.4 Image-based classification and accuracy calculation

To validate the results of the dose-response test, a follow-up validation test was conducted to evaluate whether spectral imaging could effectively discriminate between glufosinate-resistant and susceptible soybean plants. Two approaches were employed using the same spectral images: visual inspection by naked eye and machine learning (ML)-based classification using the extracted spectral indices. For visual inspection, thirteen independent evaluators with no prior experience in image analysis classified soybean plants into resistant or susceptible categories based on RGB, IR thermal, and CF images taken at 4, 6, 24, and 48 HAT. To avoid confusion caused by inverse pseudo-color scales between CF and IR thermal images, each image type was presented in a separate block with randomized order within blocks. Using the same set of images, clustering analysis was performed with the unsupervised ML algorithm Partitioning Around Medoids (PAM), based on key spectral indices such as NDI, leaf temperature, and NPQ in R 3.2.3 (R Foundation for Statistical Computing, Austria). The number of clusters was set to two, corresponding to resistant and susceptible categories. Following PAM clustering, each cluster was assigned as resistant or susceptible based on the spectral responses observed in the dose-response test.

The classification results of each method were compared against ground-truth data, which were obtained through PCR validation for glufosinate resistance as described by Yook et al. (2021). Classification accuracy was calculated as follows (Equation 9):

(9) $$\text{Accuracy}=\frac{TP+TN}{TP+TN+FP+FN}$$

where TP (true positive) and TN (true negative) indicate correctly classified resistant and susceptible soybean plants, respectively, while FP (false positive) and FN (false negative) represent misclassified individuals.

Additionally, the performance of 34 supervised ML algorithms was evaluated using the Classifier Learner app in MATLAB 2021b (**Supplementary Table 1**), based on NDI, temperature, and NPQ. A total of 96 soybean plants were used for model development, consisting of 63 glufosinate-resistant and 33 glufosinate-susceptible plants. The dataset was randomly split into training, validation, and test sets at the ratio of 7:2:1, resulting in 68, 19, and 9 plants, respectively. Five-fold cross-validation repeated 10 times on the training set for model training and hyperparameter optimization, while the independent test set was used only for the final performance evaluation. Details are provided in **Supplementary Table 2**.

### 2.5 Statistical analysis

RGB and CF indices were normalized by dividing the values of glufosinate-treated soybean plants by the mean value of the untreated control group of the same variety at the corresponding time of evaluation. All spectral indices were initially subjected to three-way repeated measures analysis of variance (RM-ANOVA), with time as the within-subject factor and herbicide dose and soybean variety (resistant and susceptible) as between-subject factors, to evaluate their main and interaction effects. Greenhouse–Geisser correction was applied when the assumption of sphericity was violated. Pairwise comparisons between soybean varieties were performed using Fisher’s LSD method when significant effects were detected. Additionally, paired t-tests were used to compare classification accuracy between visual inspection by the naked eye and ML-based classification methods at each time point. All statistical analyses were conducted by SPSS (Version 29; IBM Corp., USA) and R 3.2.3.

## 3 Results

### 3.1 Overall spectral responses of glufosinate-resistant and susceptible soybeans to glufosinate treatment

**Figure 2:**
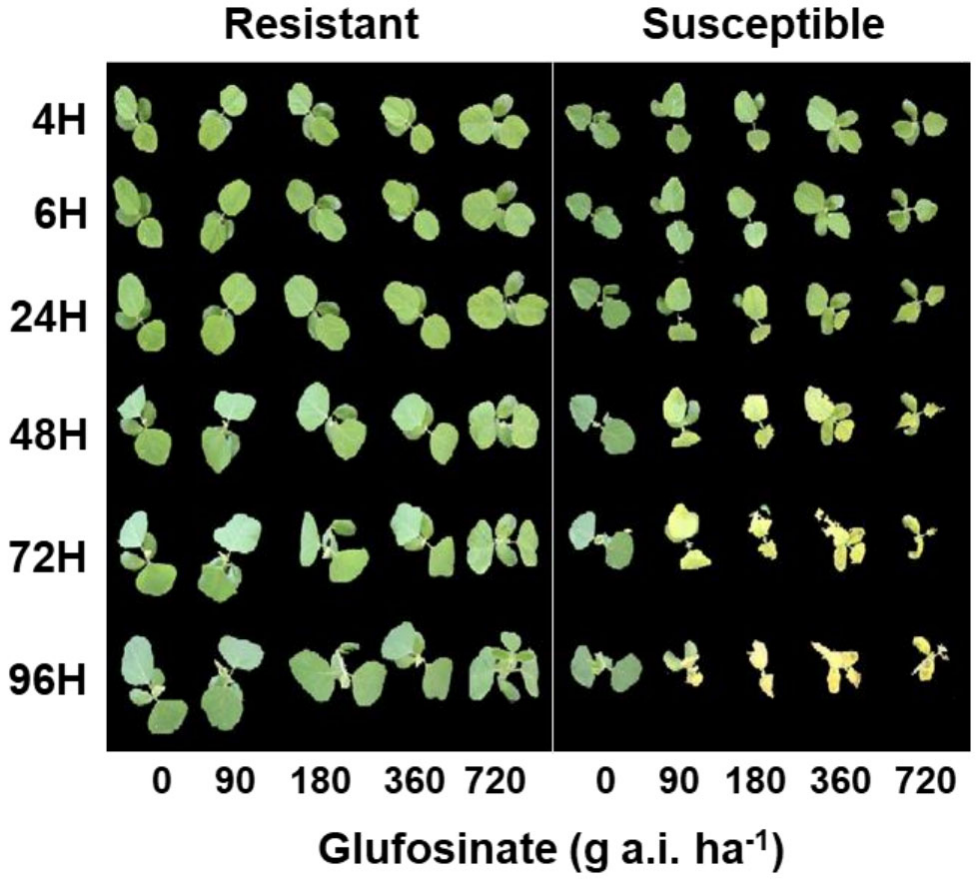
RGB images of glufosinate-resistant and susceptible soybeans over time after treatment with glufosinate at 0, 90, 180, 360, and 720 g a.i. ha^-1^.

**Figure 3:**
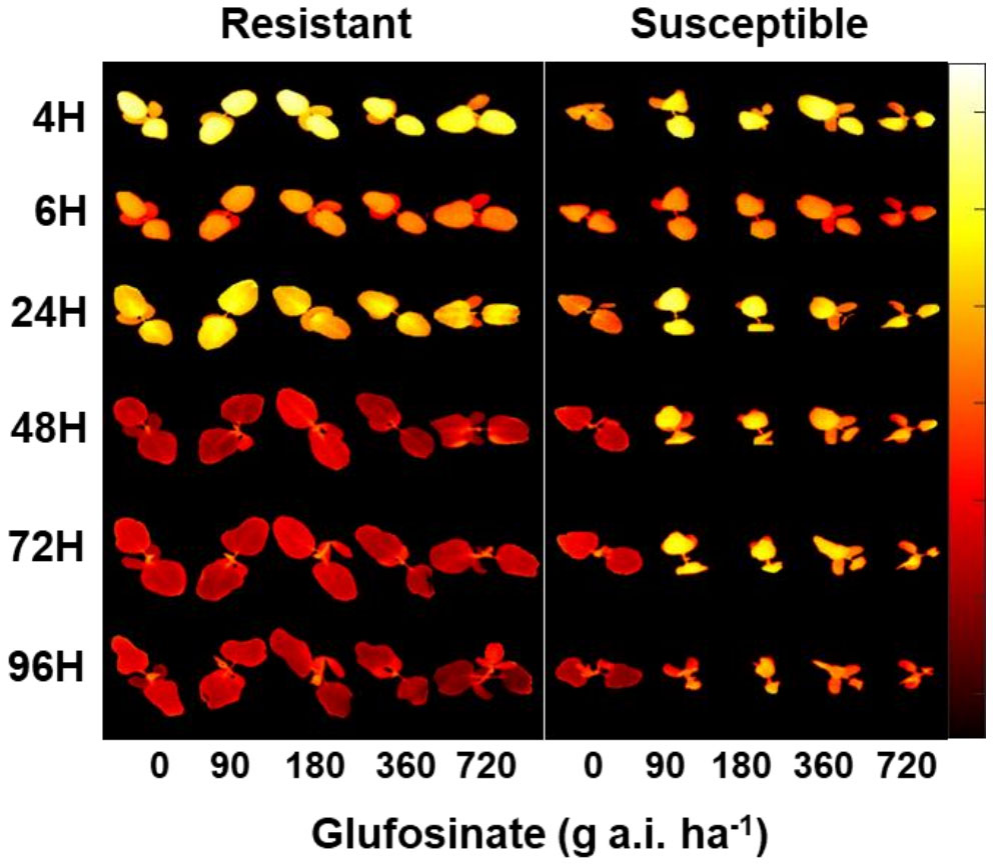
IR thermal images of glufosinate-resistant and susceptible soybeans over time after treatment with glufosinate at 0, 90, 180, 360, and 720 g a.i. ha^-1^. The color scale bar on the right represents plant temperature ranging from 30°C (black) to 40°C (white).

**Figure 4:**
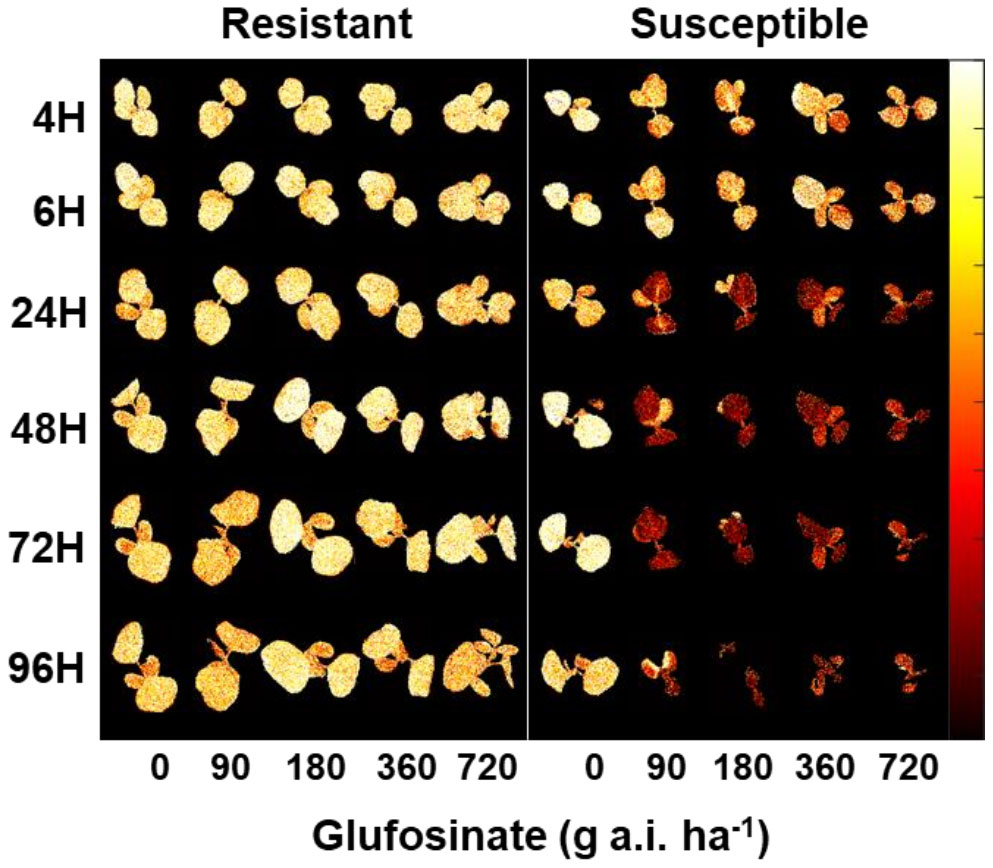
CF images of glufosinate-resistant and susceptible soybeans over time after treatment with glufosinate at 0, 90, 180, 360, and 720 g a.i. ha^-1^. The color scale bar on the right represents NPQ values ranging from 0 (black) to 1 (white).

**Figure 5:**
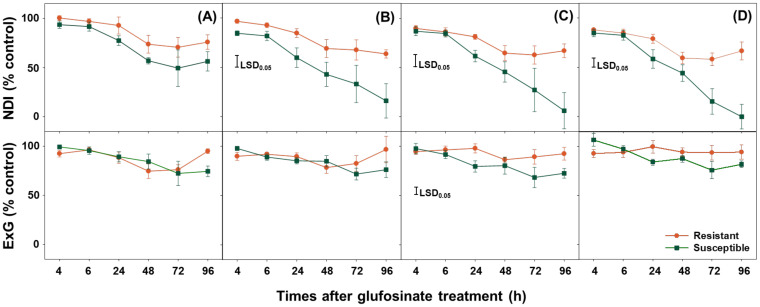
Changes in % control of NDI and ExG in glufosinate-resistant (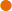) and susceptible (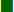) soybeans at a range of glufosinate doses, 90 **(A)**, 180 **(B)**, 360 **(C)**, and 720 **(D)** g a.i. ha^-1^. Points and error bars indicate means and standard errors of four replicates. Bars at the bottom of each plot represent the least significant difference at *P* < 0.05 and are shown only when a statistically significant difference between varieties was detected.

**Figure 6:**
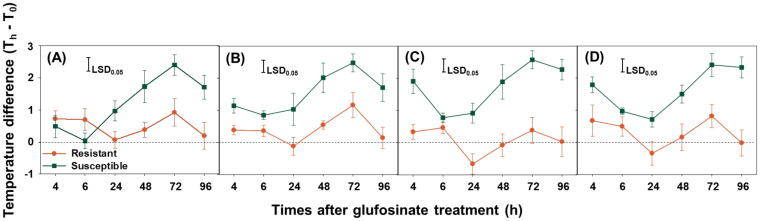
Changes in temperature difference of glufosinate-resistant (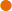) and susceptible (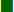) soybeans at a range of glufosinate doses, 90 **(A)**, 180 **(B)**, 360 **(C)**, and 720 **(D)** g a.i. ha^-1^. Points and error bars indicate means and standard errors of four replicates. Bars at the upper left corner represent the least significant difference at *P* < 0.05.

**Figure 7:**
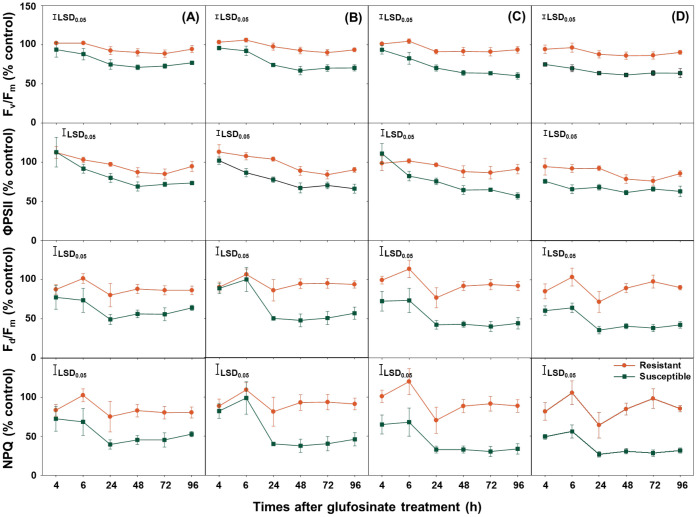
Changes in % control of CF indices, including F_v_/F_m_, Φ_PSII_, F_d_/F_m_ and NPQ, in glufosinate-resistant (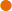) and susceptible (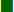) soybeans at a range of glufosinate doses, 90 **(A)**, 180 **(B)**, 360 **(C)**, and 720 **(D)** g a.i. ha^-1^. Points and error bars indicate means and standard errors of four replicates. Bars at the upper left corner represent the least significant difference at *P* < 0.05.

Spectral images acquired in the dose-response test—RGB, IR thermal, and CF images— showed different visual response patterns between glufosinate-resistant and susceptible soybeans after glufosinate treatment (**Figures 2**–**4**). These differences became more evident over time, particularly at higher glufosinate doses. Among the image types, IR thermal and CF imaging detected differences earlier than RGB imaging even at the lowest glufosinate dose of 90 g a.i. ha^-1^. Spectral indices extracted from these images also quantified differences between resistant and susceptible soybeans, with varied discrimination performance depending on dose, time, and index (**Figures 5**–**7**). A three-way RM-ANOVA revealed significant main effects of soybean variety, herbicide dose, and time on all spectral indices (*P* < 0.001, except for the effect of variety on ExG where *P* < 0.05; **Table 1**). Considering the *F* values for the variety effect, differences in IR thermal and CF indices between varieties were greater than those in RGB indices. No significant interactions among the three factors were detected for any spectral index. Significant time × variety interactions were detected for all indices (*P* < 0.05), reflecting that difference between varieties changed over time. Variety × dose interactions were significant for IR thermal and CF indices (*P* < 0.01), but not for RGB indices.

**Table 1:** Summary of three-way repeated measures ANOVA showing *F* values and significance levels for spectral indices of soybean after glufosinate treatment.

Spectral index	RGB		IR	CF			
	NDI	ExG	Temperature difference	F_v_/F_m_	Φ_PSII_	F_d_/F_m_	NPQ
Variety (V)	21.841^***^	5.353^*^	63.287^***^	116.933^***^	72.254^***^	110.405^***^	102.401^***^
Dose (D)	14.704^***^	7.509^***^	9.414^***^	22.763^***^	21.051^***^	17.142^***^	16.598^***^
V × D	1.674*^NS^*	1.033*^NS^*	5.462^**^	8.352^***^	4.996^**^	8.582^***^	7.791^***^
Time (T)	72.824^***^	12.255^***^	22.129^***^	31.350^***^	25.721^***^	16.018^***^	15.494^***^
T × V	12.525^***^	6.756^***^	11.249^***^	3.760^*^	3.116^*^	4.285^**^	3.062^*^
T × D	5.308^***^	1.086*^NS^*	2.427^**^	2.794^**^	2.434^*^	1.377*^NS^*	1.331*^NS^*
T × V × D	1.664*^NS^*	0.938*^NS^*	1.356*^NS^*	0.679*^NS^*	0.855*^NS^*	0.728*^NS^*	0.649*^NS^*

Greenhouse–Geisser correction applied for within-subject effects.

Level of significance *P < 0.05; **P < 0.01; ***P < 0.001; *NS*, not significant.

**Figure 8:**
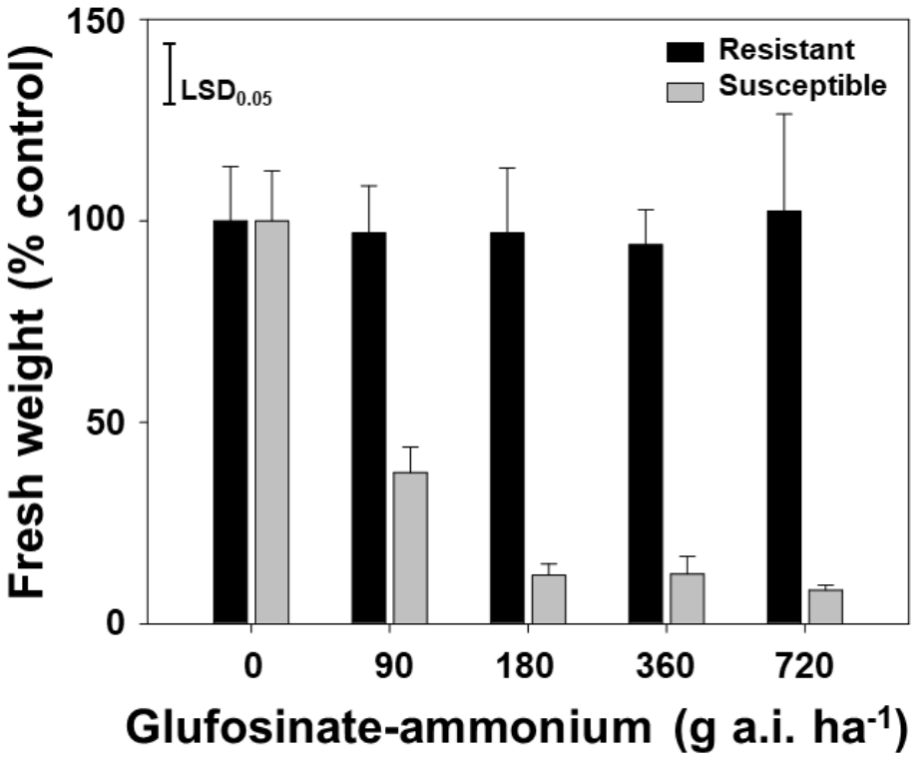
Aboveground biomass of glufosinate-resistant and susceptible soybeans at 10 days after treatment with glufosinate at 0, 90, 180, 360, and 720 g a.i. ha^-1^. The bar in the upper left corner represents the least significant difference at *P* < 0.05 for the comparison between varieties.

Aboveground biomass measured at 10 days after glufosinate treatment showed clear dose-dependent growth suppression in susceptible soybeans, whereas resistant soybeans maintained their biomass levels (**Figure 8**). These results support the idea that the early spectral differences between resistant and susceptible soybeans observed in RGB, IR thermal, and CF images correspond to long-term physiological outcomes.

### 3.2 Changes in RGB spectral responses to glufosinate

Both glufosinate-resistant and susceptible soybeans showed no visible changes in RGB images until 24 HAT. From 48 HAT, susceptible soybean leaves began to turn yellow across all glufosinate doses, with increasing chlorosis and necrosis with dose over time. Meanwhile, resistant soybean plants still remained green and largely unaffected even at the highest dose throughout the experimental period (**Figure 2**).

To quantify these differences, NDI and ExG were analyzed, and they exhibited distinct response patterns (**Figure 5**). In the susceptible soybeans, NDI decreased sharply, with higher glufosinate doses inducing a more rapid and strong response. At 96 HAT, NDI was reduced by approximately 43.8% at the 90 g a.i. ha^-1^ glufosinate treatment compared to the untreated control, and completely diminished (100% reduction) at the highest dose of 720 g a.i. ha^-1^. In contrast, NDI of the resistant soybeans also decreased after glufosinate treatment, but the reduction was minimal, ranging from 24.2% to 36.4% at 96 HAT. NDI values in both soybean varieties decreased over time after glufosinate treatment, but due to difference in response intensity, resistant and susceptible soybeans could be distinguished at most doses. NDI significantly differentiated the two varieties at 180 to 720 g a.i. ha^-1^, with the differences detected from 24 HAT and most pronounced at 96 HAT. ExG, on the other hand, showed a statistically significant difference between resistant and susceptible soybeans only at 360 g a.i. ha^-1^ glufosinate treatment (*P* < 0.05). Although ExG values of susceptible soybeans showed a slow declining trend over time, no significant difference was observed between resistant and susceptible soybeans at other doses. Even at the highest dose of 720 g a.i. ha^-1^, the difference was no longer statistically significant, indicating that ExG is a less reliable index for distinguishing resistant and susceptible soybeans after glufosinate treatment.

### 3.3 Changes in IR thermal spectral responses to glufosinate

IR thermal imaging was used to assess temperature variations in glufosinate-resistant and susceptible soybeans following glufosinate treatment (**Figure 3**). In susceptible soybeans, temperature changes relative to the untreated control were observed across glufosinate doses, even at early time points before visual symptoms appeared in RGB images (**Figures 2**, **3**). Up to 24 HAT, subtle variations in pseudo-color were observed in susceptible soybeans, while leaf temperature markedly increased thereafter. Conversely, resistant soybeans maintained relatively stable pseudo-color regardless of glufosinate treatment, indicating minimal temperature changes.

Regarding the spectral index extracted from the IR thermal images, the overall temperature difference between the untreated control and glufosinate-treated plants was greater in susceptible soybeans than in resistant soybeans (**Figure 6**). Although fluctuations were observed depending on the measurement time, temperature difference effectively distinguished resistant and susceptible soybeans at all doses. At lower doses of 90 and 180 g a.i. ha^-1^ glufosinate treatment, no significant difference between resistant and susceptible soybeans was observed in the early stages after treatment; however, differentiation became possible from 24 HAT. At higher doses, significant differences between the two varieties were detected more quickly as early as 4 HAT. Particularly, after 24 HAT, the temperature difference in susceptible soybeans averaged 1.79°C across all doses, whereas it remained at 0.22°C in resistant soybeans, showing a clear distinction. At 6 HAT, however, no significant difference was detected between resistant and susceptible soybeans at any dose. At this time point, minimal temperature differences were also observed, likely because the 6 HAT images were taken at night when glufosinate was less effective. Nevertheless, the temperature difference in susceptible soybeans generally tended to be larger than that in resistant soybeans.

### 3.4 Changes in CF spectral responses to glufosinate

CF imaging visualized differential responses of glufosinate-resistant and susceptible soybeans following herbicide treatment (**Figure 4**). Resistant soybeans exhibited minor changes in CF response across glufosinate doses and times after treatment. Although slight variations were observed, the overall pseudo-color remained bright, indicating stable physiological conditions in resistant soybeans. In contrast, susceptible soybeans rapidly darkened as both glufosinate dose and time increased, reflecting photosynthetic inhibition. In particular, clear differences in pseudo-color between the untreated control and glufosinate-treated susceptible soybeans were detected as early as 4 HAT, even before visual symptoms appeared in RGB images.

Four CF indices extracted from CF images, such as F_v_/F_m_, Φ_PSII_, F_d_/F_m_, and NPQ, quantified the differential responses of glufosinate-resistant and susceptible soybeans (**Figure 7**). In susceptible soybeans, all four CF indices showed substantial decreases, whereas resistant soybeans maintained consistently higher values.

For F_v_/F_m_, significant differences between resistant and susceptible soybeans were observed across all glufosinate doses and most time points. In particular, the difference was detected from as early as 4 HAT at the highest dose and from 6 HAT at the other doses. In susceptible soybeans, F_v_/F_m_ decreased by up to 28.9% at the lowest dose, while reductions of 33.0%, 39.7%, and 38.6% were observed at 180, 360, and 720 g a.i. ha^-^¹ glufosinate treatments, respectively. Even in resistant soybeans, F_v_/F_m_ slightly declined over time, with a maximum reduction of 14.0%. Φ_PSII_ exhibited a response pattern similar to that of F_v_/F_m_, with significant differentiation detected across all glufosinate doses and most time points. However, at the highest dose, significant differentiation was possible from 6 HAT rather than 4 HAT. Non-photochemical indices, F_d_/F_m_ and NPQ, revealed distinct trends as well. Although fluctuations were observed in F_d_/F_m_ and NPQ, resistant soybeans consistently maintained higher values, whereas susceptible soybeans exhibited a drastic decline. In susceptible soybeans, F_d_/F_m_ and NPQ values sharply decreased within 24 hours. Subsequently, F_d_/F_m_ declined to 35.5% and NPQ to 26.7%, maintaining significantly lower values compared with the minimum values of resistant soybeans (71.7% and 64.1%, respectively). F_d_/F_m_ and NPQ effectively differentiated the two varieties from 24 HAT at lower doses of 90 and 180 g a.i. ha^-1^, whereas at higher doses, significant differences were detected as early as 4 HAT. Among the four CF indices, F_v_/F_m_ differentiated the two varieties earliest at lower doses of 90 and 180 g a.i. ha^-1^. At higher doses of 360 and 720 g a.i. ha^-1^, NPQ exhibited the earliest differentiation and the clearest difference between the two varieties.

### 3.5 Discrimination of glufosinate resistance in mixed plantings using spectral imaging

**Figure 9:**
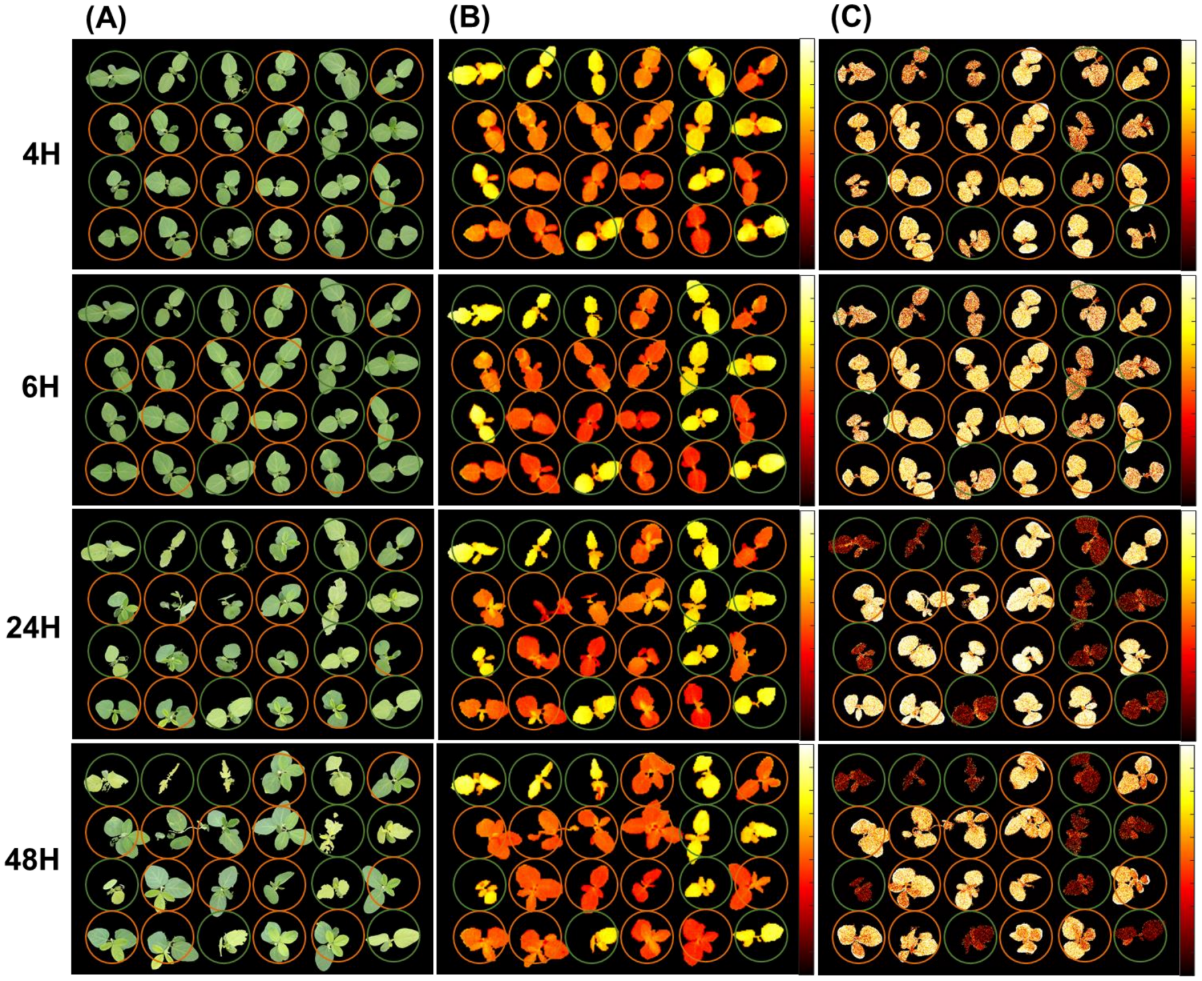
RGB **(A)**, IR thermal **(B)**, and CF **(C)** images of glufosinate-resistant (orange circles) and susceptible (green circles) soybean individuals at 4, 6, 24, and 48 hours after treatment with 180 g a.i. ha^-1^ glufosinate. Circle colors were assigned according to PCR validation results. Scales indicate plant temperature **(B)** and NPQ gradient **(C)**.

**Figure 10:**
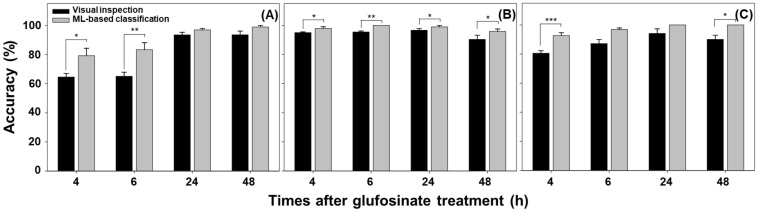
Classification accuracy at different time points after glufosinate treatment, based on RGB **(A)**, IR thermal **(B)**, and CF **(C)** images in mixed plantings of resistant and susceptible soybeans. Accuracy was evaluated through visual inspection by naked eye and machine learning (ML)-based classification using the PAM algorithm. Error bars indicate standard errors of the mean (SE). Asterisks indicate significant differences between the two approaches (paired t-test, * *P* < 0.05; **P < 0.01; ***P < 0.001).

Based on the results of the dose-response test, a glufosinate treatment of 180 g a.i. ha^-1^ was selected for the validation test. This was the lowest glufosinate dose that enabled discrimination between resistant and susceptible plants across RGB, IR thermal, and CF indices. Spectral images acquired from soybeans treated at this dose also successfully classified individual plants into resistant or susceptible categories (**Figures 9**, **10**).

For visual inspection by the naked eye, classification accuracy tended to increase over time, with the highest performance observed in IR thermal images (96.6%), followed by CF (94.2%) and RGB images (93.6%). However, no image type reached 100% accuracy at any time point. By image type, RGB images showed relatively low accuracy at early time points (64.5% at 4 HAT), but performance improved significantly after 24 HAT, exceeding 93%. IR thermal images showed consistently high accuracy, maintaining over 95% from as early as 4 HAT, with only a slight decline at 48 HAT. CF image-based accuracy reached 80.6% at 4 HAT and 94.2% at 24 HAT. The classification accuracy of 13 individual evaluators ranged from 73.3% to 93.0% depending on their subjective classification criteria for discrimination between glufosinate-resistant and susceptible soybeans.

ML-based classification using the PAM algorithm distinguished resistant and susceptible soybeans faster and more accurately than visual inspection by the naked eye. Among the spectral indices, NDI derived from RGB images showed a steady increase in classification accuracy, from 77.1% at 4 HAT to 97.9% at 48 HAT. Leaf temperature extracted from IR thermal images enabled early classification, achieving 100% accuracy as early as 6 HAT and maintaining over 95% accuracy at all time points. NPQ derived from CF images showed a high accuracy of 92.7% at 4 HAT, which continued to improve and reached 100% after 24 HAT.

## 4 Discussion

### 4.1 Early detection of glufosinate-resistant soybean using spectral imaging

Spectral imaging technologies enable the detection of herbicide-induced responses within hours after treatment, whereas conventional diagnostic methods generally require several days to assess herbicide injury (Weber et al., 2017; Ali et al., 2022; Jeong et al., 2024). In this study, spectral indices derived from RGB, IR thermal, and CF images distinguished between glufosinate-resistant and susceptible soybeans as early as 4 to 24 HAT. IR thermal and CF indices detected differences before visual symptoms appeared in RGB images (**Table 1**; **Figures 2**–**7**). These findings highlight the potential applicability of spectral imaging as an early and non-destructive tool for identifying glufosinate-resistant soybean plants. Furthermore, the consistency between early spectral responses and the reduction in aboveground biomass measured 10 days after glufosinate treatment supports their reliability for early glufosinate resistance diagnosis (**Figure 8**).

Jones et al. (2023) reported that the green leaf index derived from multispectral images of leaf discs could be used to distinguish between glufosinate-resistant and susceptible soybeans at 48 HAT. By extending this approach, this study demonstrated that whole-plant spectral imaging could identify glufosinate resistance earlier and non-destructively. Especially, the use of IR thermal and CF images enabled further enhancement for more rapid and accurate classification. The ability to detect early changes in spectral images, which reflect physiological responses to glufosinate, provides advantages in terms of time efficiency and labor savings for monitoring in weed management.

### 4.2 Performance comparison of spectral indices from RGB, IR thermal, and CF imaging

All three spectral imaging types used in this study (RGB, IR thermal, and CF imaging) differed in sensitivity and response timing when comparing glufosinate-resistant and susceptible soybeans. These differences are related to the mode of action (MoA) of glufosinate. In susceptible plants, glufosinate inhibits glutamine synthetase, disrupting key physiological processes and causing ammonia accumulation, reducing photosynthesis, and increasing ROS production. These changes ultimately lead to cellular damage (Takano and Dayan, 2020). IR thermal and CF imaging can detect changes earlier than RGB imaging, as both techniques capture spectral changes that reflect early physiological responses such as altered leaf temperature and photosynthetic inhibition. In contrast, RGB imaging focuses on visible injury that appears later.

IR thermal imaging revealed a significant increase in leaf temperature in susceptible soybeans following glufosinate treatment (**Figures 3**, **9B**). These changes were associated with photosynthetic inhibition due to stomatal closure and reduced transpiration and/or oxidative stress-induced cell damage (Pineda et al., 2021). Consistently, similar increases in leaf temperature following glufosinate treatment have been reported in other plant species (Takayama and Omasa, 2005; Jeong et al., 2024). However, leaf temperature is affected by multiple environmental factors, including soil moisture, air temperature, and relative humidity, and therefore does not exclusively reflect herbicide-induced stress (Pineda et al., 2021). For this reason, we used the temperature difference compared to untreated controls as a normalized index to evaluate thermal responses across doses and time points. In the dose-response test, although fluctuations were observed, the temperature difference in susceptible soybeans remained significantly higher than that of resistant soybeans at most glufosinate doses (**Figure 6**). At 6 HAT, however, differences between resistant and susceptible soybeans were minimal across all doses, likely because the images were taken at night, when glufosinate activity is reduced due to its light-dependent MoA. This is consistent with previous reports that glufosinate-induced ROS production requires light and is suppressed in the dark (Takano et al., 2020). This suggests that IR thermal imaging is more reliable under light conditions, where herbicidal activity is fully expressed. Accordingly, in the validation test, all IR thermal images were acquired during the light phase. Under these conditions, leaf temperature was validated as a strong index for distinguishing between glufosinate-resistant and susceptible soybeans at single time points after herbicide treatment (**Figure 9**). For instance, ML-based classification using IR thermal imaging achieved 100% accuracy as early as 6 HAT (**Figure 10**). These findings demonstrate the effectiveness of thermal response as a key indicator for early detection of glufosinate resistance.

CF imaging has been widely used to assess photosynthetic responses of plants to various stresses including herbicides, which directly or indirectly inhibit photosynthesis. Previous studies have established the effectiveness of CF analysis as a rapid and non-invasive tool for evaluating herbicidal effects (Vítek et al., 2020; Jeong et al., 2024; Noh et al., 2025), assessing crop injury (Li et al., 2017), and identifying herbicide-resistant weeds or crops (Wang et al., 2016; Zhang et al., 2016; Weber et al., 2017; Zhang and Kim, 2018), under controlled and field conditions. Among CF indices, F_v_/F_m_ showed a consistent response in this study. It effectively distinguished between resistant and susceptible soybeans at all doses and most time points following glufosinate treatment. This is likely due to its low variability among replicates, even when differences between resistant and susceptible soybeans were small (**Figure 7**). In susceptible soybeans, F_v_/F_m_ values, which reflect the maximum photochemical efficiency of PSII, gradually decreased after glufosinate treatment. NPQ dropped sharply between 6 and 24 HAT and remained distinct from resistant soybeans, indicating disruption of non-photochemical energy dissipation under glufosinate-induced stress. Similar response patterns have been reported in *Chenopodium album* and *Lemna minor*, supporting that NPQ is a sensitive indicator of early herbicide-induced stress (Frankart et al., 2003; Takano et al., 2020). In short, F_v_/F_m_ and NPQ extracted from CF images can identify resistant soybeans within hours of glufosinate treatment. This indicates that CF imaging is suitable for the early identification of herbicide resistance. However, the requirement for dark adaptation before image acquisition may limit its practical use, and therefore utilizing CF indices such as Φ_PSII_ that can be measured under light-adapted conditions would be beneficial.

RGB imaging is useful for detecting changes in plant color and morphology caused by herbicide treatment. As the most accessible imaging method, high-resolution RGB images can be easily obtained using digital cameras, inexpensive UAV-based RGB sensors, or even smartphones (Chung et al., 2018; Abrantes et al., 2021). However, compared to IR thermal and CF imaging, RGB imaging is less responsive to stress in the early phases. Nonetheless, RGB images are still informative, and in particular, extracted vegetation indices can provide objective and quantitative measurements compared to conventional visual scoring (Ali et al., 2013; Marques et al., 2021). In previous studies, NDI and ExG effectively reflected plant discoloration caused by herbicides with different MoAs (Jeong et al., 2024; Noh et al., 2025). Similarly, in this study, both NDI and ExG gradually decreased after glufosinate treatment in glufosinate-susceptible soybeans, and the decrease in NDI was much greater, while these indices remained relatively stable in resistant soybeans. As a result, NDI distinguished the two varieties at 180 to 720 g a.i. ha^-1^, with the difference detected from 24 HAT and most evident at 96 HAT, but ExG failed to consistently differentiate between them (**Figure 5**). These findings highlight NDI as a more sensitive vegetation index for glufosinate resistance detection.

While the timing of discrimination between resistant and susceptible soybeans using spectral indices varied slightly depending on the glufosinate dose, CF and IR thermal indices generally showed significant differences between the two varieties as early as 4 HAT, particularly at higher glufosinate doses. This demonstrates their superior diagnostic performance compared to RGB indices. Although less sensitive in the early phase, RGB imaging may serve as a practical supplement, particularly when CF and IR thermal imaging systems are not available.

### 4.3 Validation of image-based classification in mixed planting of resistant and susceptible soybeans

To validate the utility of spectral imaging assessed in the dose-response test, a validation test was performed using mixed-planted soybeans consisting of resistant and susceptible individuals. For this test, we selected three key spectral indices (NDI, leaf temperature, and NPQ) that previously showed clear differences between glufosinate-resistant and susceptible soybeans. These indices again demonstrated high classification accuracy, confirming their consistency and reliability across different genetic backgrounds. Moreover, classification was achieved without normalization against untreated controls, demonstrating the practical value of this method for detecting glufosinate-resistant individuals. The mixed planting soybeans employed in this test were genetically heterogeneous, derived from interspecific crosses between GM and wild soybean, suggesting the potential applicability of this approach across broader genetic backgrounds.

To further assess practical applicability, we employed several approaches including visual inspection and ML-based classification. Despite differences in performance and processing among the classification approaches, spectral imaging consistently identified glufosinate-resistant soybeans accurately. Although slightly less accurate, visual inspection still showed high classification accuracy and can be used as a practical option for rapid, on-site evaluation immediately after image acquisition. The clear pseudo-color contrast in IR thermal images improved visual clarity, facilitating rapid and accurate classification by human evaluators (**Figures 9**, **10**). However, performance varied among evaluators, reflecting the subjective nature of human visual evaluation. For ML-based classification using PAM, spectral indices extracted from the same spectral images used for visual inspection by the naked eye were employed. This approach consistently outperformed visual inspection across all time points, especially during early stages, showing statistically higher accuracy (**Figure 10**). This method offers objective and repeatable results with minimal data processing and exhibits less variability than the subjective classifications made by human evaluators. However, as the number of clusters must be predefined (e.g., two in this study), this approach is applicable only when both resistant and susceptible individuals coexist. Additionally, performance may be limited when resistant individuals are rare. In such cases, supervised ML approaches may provide an alternative because they do not require predefined cluster structures.

To explore more advanced classification strategies, we further evaluated supervised ML models. Five-fold cross-validation generally showed high classification performance, although classification accuracy varied among algorithms and spectral indices. IR thermal and CF indices showed higher and earlier classification performance than RGB index for most evaluated algorithms (**Supplementary Figure S1**). Complete performance statistics for all evaluated models are provided in **Supplementary Data Sheet 1**. In particular, leaf temperature extracted from IR thermal images enabled accurate discrimination between resistant and susceptible soybeans at early assessment times after glufosinate treatment. For example, leaf temperature and NPQ achieved 100% mean CV accuracy as early as 6 HAT and 24 HAT, respectively, using the Fine Tree classifier (**Supplementary Figure S2**; **Supplementary Table 3**). These findings further support the reproducibility of the index-specific differences observed in the dose-response and validation tests described above and demonstrate the potential of image-based ML approaches. However, considering the relatively small dataset used in this study, further validation using larger and more diverse populations is needed. These approaches may be more suited for large-scale applications such as high-throughput screening platforms (Nagasubramanian et al., 2022).

Taken together, these results support the reproducibility and practical value of spectral imaging, particularly IR thermal imaging, for early and reliable detection of glufosinate resistance. Depending on the circumstance and available resources, different approaches can be adopted including visual inspection, unsupervised clustering, or supervised ML-based classification. Further validation across diverse genotypes and field conditions is essential to expand the utility of this approach.

### 4.4 Further applications for monitoring gene flow and herbicide-resistant weeds

This study provides practical insights into monitoring transgene flow from herbicide-resistant GM soybean and the early detection of glufosinate resistance. Conventional methods for detecting plants containing transgenes, such as PCR and lateral flow protein strip tests, have been widely used (Lee et al., 2021; Zeng et al., 2021). While they are accurate, these methods are destructive, costly, time-consuming, and require specialized equipment and skilled technicians. In contrast, the image-based identification method developed in this study provides a rapid and non-destructive screening approach that can complement conventional methods. As a result, it is highly suitable for practical applications such as identifying transgene-containing plants after the accidental release of glufosinate-resistant GM soybean or screening progeny in gene flow experiments. In such situations, early diagnosis enables timely isolation or removal of transgenic individuals to minimize unintended gene flow. In addition, this approach could be expanded to detect herbicide-resistant weeds. Early spectral changes associated with physiological responses captured by CF and IR thermal imaging may also be useful for high-throughput and objective identification of herbicide-resistant weeds, as demonstrated in previous studies (Jeong et al., 2024; Noh et al., 2025). This provides a viable alternative to conventional whole-plant assays, which are labor-intensive and time-consuming. However, since this study was conducted under controlled growth chamber conditions, further verification under various environmental conditions is required. Leaf temperature is known to be influenced by multiple environmental factors, including air temperature, relative humidity, vapor pressure deficit, soil water status, radiation intensity, and canopy architecture. Therefore, the thermal response identified in this study may not be directly transferable to field environments. Nevertheless, the objective of this study was to evaluate the relative thermal responses of resistant and susceptible plants following herbicide application under standardized conditions. For practical screening applications, thermal traits may be normalized using untreated reference plants, background canopy temperatures, or temperature differentials relative to ambient air temperature. Such approaches have been used in thermal phenotyping studies to minimize environmental effects and improve the robustness of thermal measurements across variable conditions (Jones et al., 2009; Jeong et al., 2024; Noh et al., 2025). Future studies should evaluate the robustness of these normalization approaches under field conditions where environmental variability is substantially greater than in controlled environments. Overall, our findings suggest that plant spectral image analysis may serve as a useful tool for early identification of glufosinate-resistant soybean, which can be an essential component for the risk management of GM soybean cultivation.

## Data Availability

The original contributions presented in the study are included in the article/**Supplementary Material**. Further inquiries can be directed to the corresponding author.
