# Supplementary material for: Early identification of glufosinate-resistant soybeans using plant image analysis
Source: Front Plant Sci. 2026 Aug 4;17:1886688. doi: 10.3389/fpls.2026.1886688 (PMC13483073; doi:10.3389/fpls.2026.1886688)
Supplement: Supplementary file 1 [file Supplementaryfile1.docx]

**Supplementary materials**

**Table S1.** List of 34 supervised machine learning algorithms evaluated for classifying glufosinate-resistant and susceptible soybeans. All algorithms were implemented using the Classifier Learner app in MATLAB 2021b.

| Model category | Machine learning algorithm |
| --- | --- |
| Decision Tree | Fine Tree |
|  | Medium Tree |
|  | Coarse Tree |
| Discriminant Analysis | Linear Discriminant |
|  | Quadratic Discriminant |
| Logistic Regression (Binary GLM) | Logistic regression (Binary GLM) |
| Regularized Logistic Regression | Efficient Logistic Regression |
| Linear SVM | Efficient Linear SVM |
| Naive Bayes | Gaussian Naive Bayes |
|  | Kernel Naive Bayes |
| Support Vector Machine | Linear SVM |
|  | Quadratic SVM |
|  | Cubic SVM |
|  | Fine Gaussian SVM |
|  | Medium Gaussian SVM |
|  | Coarse Gaussian SVM |
| k-Nearest Neighbor | Fine KNN |
|  | Medium KNN |
|  | Coarse KNN |
|  | Cosine KNN |
|  | Cubic KNN |
|  | Weighted KNN |
| Ensembles | Boosted Trees |
|  | Bagged Trees |
|  | Subspace Discriminant |
|  | Subspace KNN |
|  | RUSBoosted Trees |
| Neural Network | Narrow Neural Network |
|  | Medium Neural Network |
|  | Wide Neural Network |
|  | Bilayered Neural Network |
|  | Trilayered Neural Network |
| Kernel | SVM Kernel |
|  | Logistic Regression Kernel |

**Table S2.** Number of glufosinate-resistant and susceptible soybean plants in the training, validation, and test datasets used for supervised machine-learning model development and evaluation.

| Dataset | Resistant | Susceptible |
| --- | --- | --- |
| Training | 45 | 23 |
| Validation | 12 | 7 |
| Test | 6 | 3 |

**Table S3.** Class-wise performance metrics of the representative Fine Tree classifier corresponding to the confusion matrices shown in Figure S2. Metrics were calculated using the independent test subset described in Table S2. Precision, recall, specificity, and F1-score were calculated separately for resistant and susceptible soybean classes.

| Spectral Index | Time (HAT) | Class | Precision | Recall | Specificity | F1-score |
| --- | --- | --- | --- | --- | --- | --- |
| NDI | 4 | Resistant | 0.71 | 0.83 | 0.33 | 0.77 |
|  |  | Susceptible | 0.50 | 0.33 | 0.83 | 0.40 |
|  | 6 | Resistant | 0.86 | 1.00 | 0.67 | 0.92 |
|  |  | Susceptible | 1.00 | 0.67 | 1.00 | 0.80 |
|  | 24 | Resistant | 1.00 | 1.00 | 1.00 | 1.00 |
|  |  | Susceptible | 1.00 | 1.00 | 1.00 | 1.00 |
|  | 48 | Resistant | 1.00 | 1.00 | 1.00 | 1.00 |
|  |  | Susceptible | 1.00 | 1.00 | 1.00 | 1.00 |
| Leaf temperature | 4 | Resistant | 1.00 | 1.00 | 1.00 | 1.00 |
|  |  | Susceptible | 1.00 | 1.00 | 1.00 | 1.00 |
|  | 6 | Resistant | 1.00 | 1.00 | 1.00 | 1.00 |
|  |  | Susceptible | 1.00 | 1.00 | 1.00 | 1.00 |
|  | 24 | Resistant | 1.00 | 1.00 | 1.00 | 1.00 |
|  |  | Susceptible | 1.00 | 1.00 | 1.00 | 1.00 |
|  | 48 | Resistant | 1.00 | 0.83 | 1.00 | 0.91 |
|  |  | Susceptible | 0.75 | 1.00 | 0.83 | 0.86 |
| NPQ | 4 | Resistant | 0.86 | 1.00 | 0.67 | 0.92 |
|  |  | Susceptible | 1.00 | 0.67 | 1.00 | 0.80 |
|  | 6 | Resistant | 0.86 | 1.00 | 0.67 | 0.92 |
|  |  | Susceptible | 1.00 | 0.67 | 1.00 | 0.80 |
|  | 24 | Resistant | 1.00 | 1.00 | 1.00 | 1.00 |
|  |  | Susceptible | 1.00 | 1.00 | 1.00 | 1.00 |
|  | 48 | Resistant | 1.00 | 1.00 | 1.00 | 1.00 |
|  |  | Susceptible | 1.00 | 1.00 | 1.00 | 1.00 |

HAT, hours after treatment; NDI, normalized difference index; NPQ, non-photochemical quenching.

**Figure S1.** Mean classification accuracy of 34 supervised machine learning algorithms at different time points after glufosinate treatment, based on NDI (A), temperature (B), and NPQ (C) in mixed plantings of resistant and susceptible soybeans. Model performance was evaluated using repeated five-fold cross-validation (10 repeats), and mean cross-validation accuracy ± standard deviation was used for comparison among algorithms.

**Figure S2.** Confusion matrices of the representative Fine Tree classifier generated using the test subset for classifying glufosinate-resistant and susceptible soybeans based on NDI, leaf temperature, and NPQ at different time points after treatment.
